# Research on the evaluation system of prosthesis structure type implanted with porous structure

**DOI:** 10.3389/frtra.2025.1528548

**Published:** 2025-05-22

**Authors:** Ye Zhu, Yong Jiang, Lei Lei, Hongchi Liu, Tianmin Guan

**Affiliations:** ^1^ School of Mechanical Engineering, Dalian Jiao Tong University, Dalian, China; ^2^ Department of Spine Surgery, Dalian Second People’s Hospital, Dalian, Liaoning, China

**Keywords:** porous structure, evaluation system, processability, bone ingrowth, implanted prosthesis

## Abstract

The porous structure can effectively reduce the stress shielding effect in the process of implanting prostheses in the treatment of bone defects, but the performance of different types of porous structures directly affects the treatment effect, so it is necessary to evaluate a variety of different porous structures and select the most suitable structure type for implantation to improve the treatment effect. Based on the three-dimensional model of porous structure, this paper uses numerical analysis to evaluate the mechanical properties of porous structure and completes the primary selection of porous structure; secondly, the indexes and weights affecting the performance of porous structure are clarified, the calculation method of evaluation value is determined, and the evaluation system of implanted prosthesis with porous structure is constructed; then, through mechanical experiments and animal experiments, the machinability index and bone ingrowth index of the primary structure and commonly used clinical structure are studied; finally, according to the evaluation system, the most suitable type of porous structure for implantation is selected. The results of this study found that the tetrahedral body-centered structure [diamond structure] is the optimal structure type for the preparation of implanted prostheses with porous structures. The implantation of tetrahedral body-heart structure is of great significance for the treatment of segmental bone defects and can improve the quality of life of patients.

## 1 Introduction

**Figure 1:**
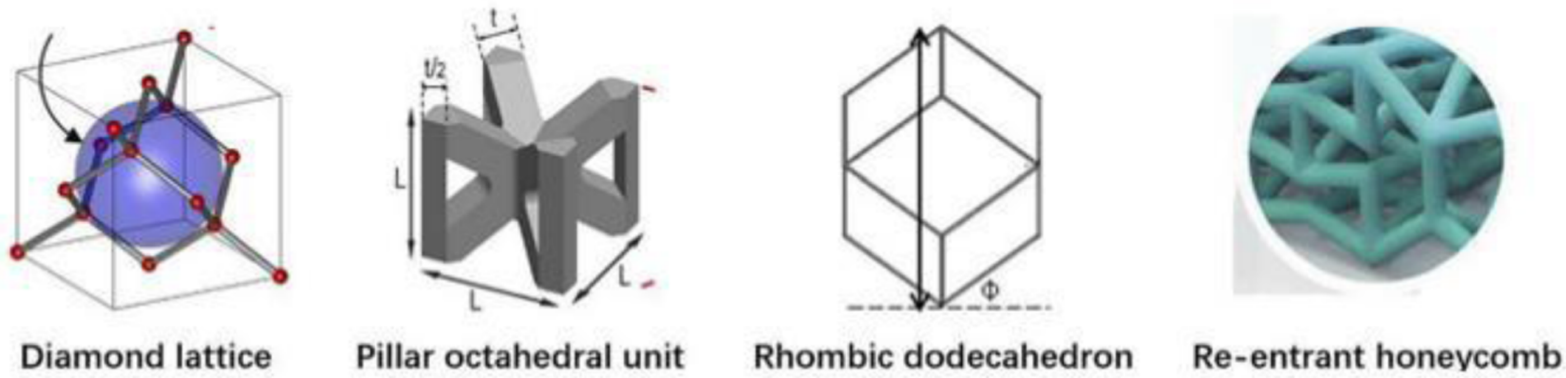
Regular porous structure for prosthesis design.

Segmental bone defects are mainly caused by high-energy trauma, infection, tumor resection, and nonunion of fractures (1, 2). Stress occlusion often occurs during the implantation of prostheses for the treatment of segmental bone defects, due to the mismatch of material properties between the implanted prosthesis and the bone (3). At present, the main approach is to make prostheses from porous metal materials, by adjusting their topology (4) and relative density to meet mechanical and biological requirements, so that material properties, such as compressive strength, stiffness, and elasticity, are similar to those of the bone at the corresponding regeneration stage (5–7). There are great differences (Figure 1) in the mechanical properties and bone ingrowth of different porous structure types, so further research is needed.

Given the large differences in the performance of different porous structure types, scholars at home and abroad have done a lot of research and exploration. In 2017, (8) studied the compressive mechanical properties of four different pore structures and found that the compressive modulus and strength of the four structures showed a downward trend according to the order of face-centered cubic structure, body-centered cubic structure, spherical hollow cubic structure, and cubic structure. In 2018, (9) designed the face-centered cube, the vertex cube, and the edge center cube, and found that the face-centered cube and vertex cube performed better mechanically than the edge center cube. In 2018, (10) summarized the advantages and disadvantages of common regular porous structure implant prosthesis, mainly including cubic structure, diamond structure, and rhombic 12hedral structure. In 2020, (11, 12) showed that the controllable porous structure matches the elastic modulus of the bone, which can reduce the stress occlusion effect and improve the long-term stability of the implanted prosthesis. Secondly, in 2014, (13) designed a square microstructure femoral stem, and the results showed that the prosthesis with a porous structure could alleviate stress occlusion, but the square hole was less isotropic. In 2017, (14) used the finite element method to study and calculate the elastic modulus of two porous structures, the body-centered cube and the enhanced body-centered cube in different directions and concluded that the body-centered cube structure is better than the enhanced body-centered cube structure in achieving isotropy. In addition, (15) noted that the human environment is highly corrosive, while biomaterials are generally biologically active and interact with the environment after implantation. Therefore, the design of the implant must be both functional and biocompatible. In 2018, (16) suggested that the interconnected pore structure is beneficial for nutrient exchange and the formation of blood vessels, which can achieve bone outgrowth and long-term biological fixation. In 2021, studies (17) showed that large aperture structures have advantages for the formation of blood vessels compared to structures with smaller pore sizes, thereby promoting bone growth. Many scholars have conducted comparative studies on the mechanical properties and bone ingrowth of a variety of porous structures, but due to the obvious differences in human bones, implantable prostheses with a single elastic modulus cannot meet the needs of all bones, and it is rare to screen out a certain type of porous structure through mechanical experiments and animal experiments to make it suitable for implanted prosthesis design. Therefore, it is necessary to study the mechanical and biological properties of a variety of different porous structures and construct an evaluation system for implanted prostheses with porous structures, to achieve the best matching between implanted prostheses and individual bones on this basis.

**Figure 2:**
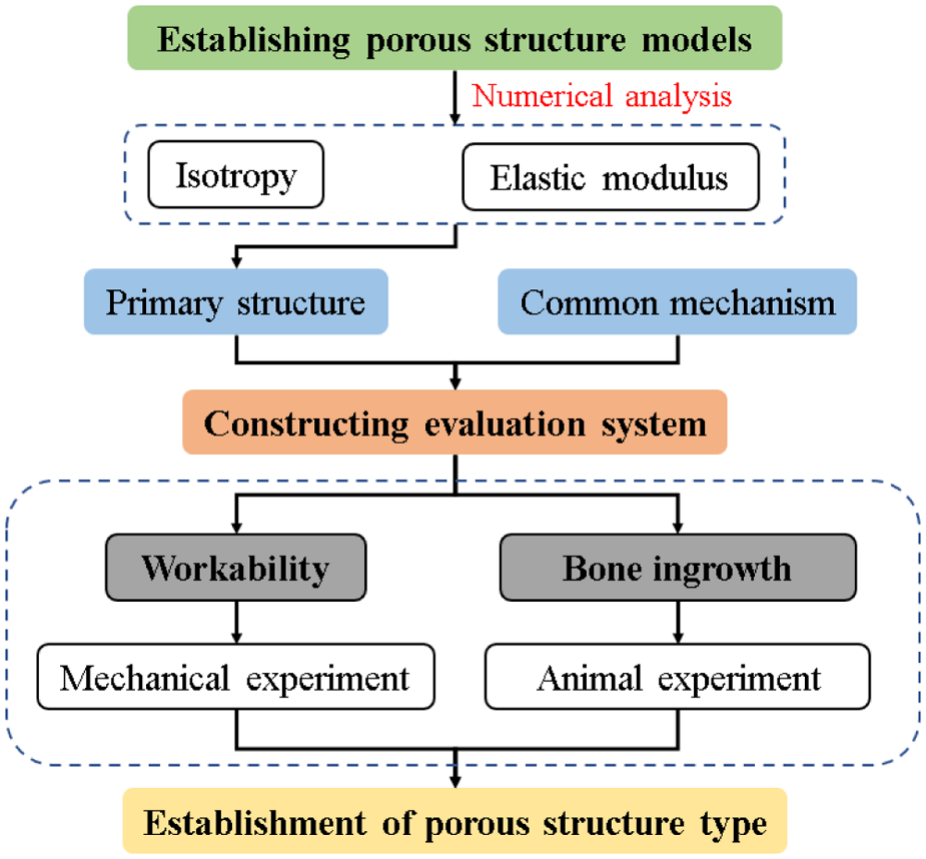
Technology route.

In this study, numerical analysis was applied to the 3D model of porous structure, and its isotropic properties were evaluated, which was used as the basis for the preliminary selection of the structure. Subsequently, a comprehensive evaluation system for implanted prostheses with porous structures was constructed. Furthermore, the mechanical properties and biocompatibility experiments were conducted to verify the processability and osseointegration indexes of the primary structure and the commonly used clinical structure. Based on the above results, the tetrahedral body-centered structure was finally identified as the preferred porous structure type for implantable prosthesis research. Figure 2 shows the technical process described in the article.

## 2 Preliminary selection of porous structure

In the design of porous implantable prostheses, the isotropic porous structure is conducive to stability, while the anisotropic structure has uncertainty and stability problems. In addition to comparing the isotropy of porous structure, it is also necessary to study its elastic modulus, so numerical analysis of porous structure can be used as a preliminary screening basis.

### 2.1 Establishment of porous structure model

**Figure 3:**
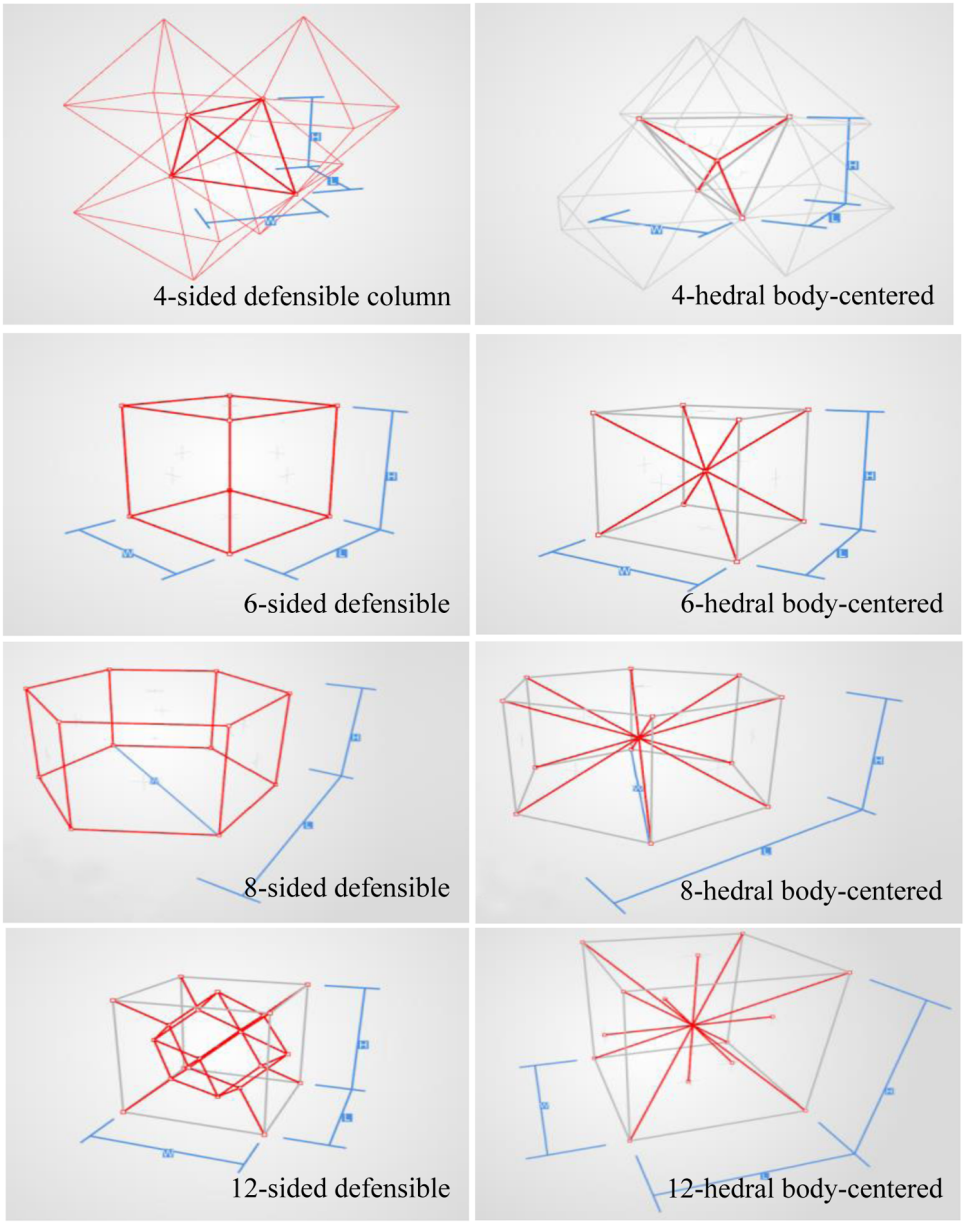
Build porous structures.

In order to analyze the mechanical properties of the porous structure, it is necessary to establish a porous structure model, and construct the porous structures of 4hededron, 6hededron, 8hedra and 12hedra according to the microstructure of the unit cell, and each spatial configuration includes a face-column structure and a body-centered structure, as shown in Figure 3.

**Figure 4:**
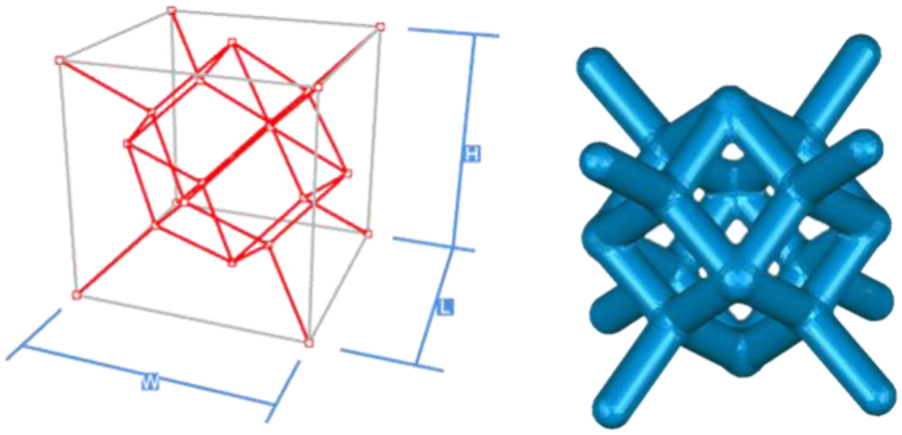
Build the cell structure of the porous structure.

**Figure 5:**
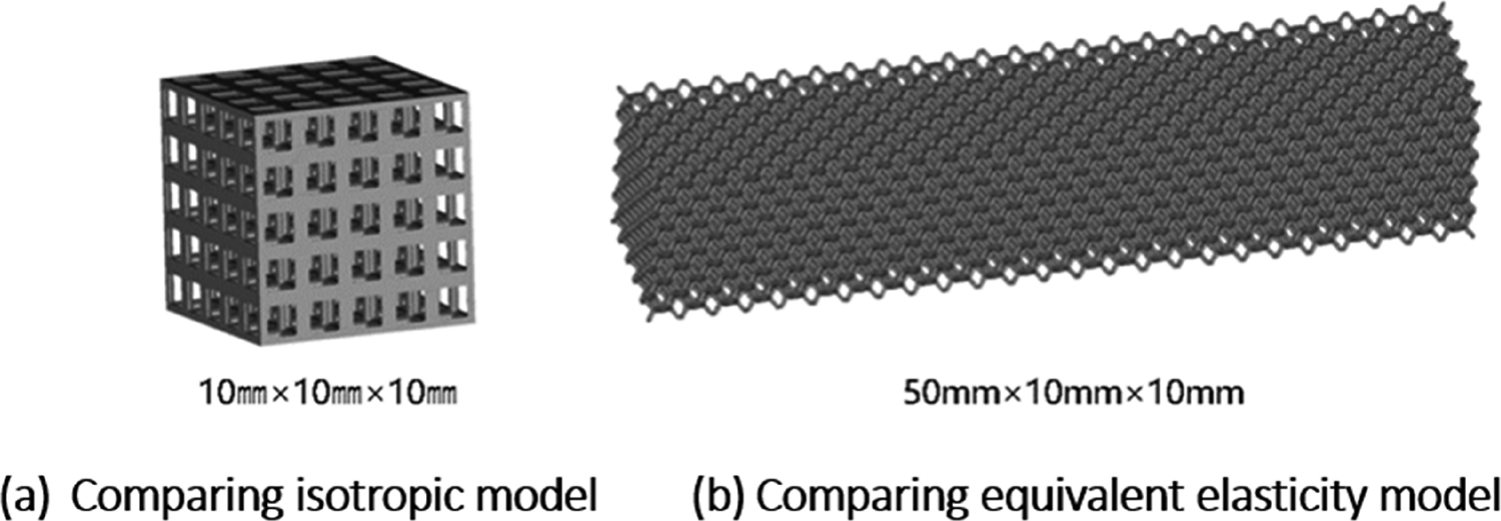
Porous structure model.

Creo software was used to construct the single unit cells of each unit cell structure, with a unit cell side length of 2 mm (W, H, and L lengths are all 2 mm in the figure), and a porosity of 80%. The specific modeling process is as follows: firstly, the lines corresponding to the vertices of the body, face, and polyhedron are made in space, and then the corresponding rod-like structure is stretched, and finally the excess structure is removed by Boolean operation to form a repeatable superimposed porous cell structure. Take the 12-hedral column structure as an example, as shown in Figure 4. As mentioned above, the appropriate equivalent medium can replace the isotropic porous structure as the research object, and the isotropic porous structure as the implantable prosthesis design can effectively avoid the trouble caused by the anisotropy of the implanted prosthesis in the treatment process of repairing bone defects. Therefore, it is necessary to study the isotropy of the porous structure, and establish a 10mm × 10mm × 10 mm static simulation test porous structure model, as shown in (a) in Figure 5.

In order to analyze the mechanical properties of different types of porous structures, it is necessary to construct the same numerical simulation mechanical model as the experiment, and use Magics software to combine the porous cell structures into 50mm × 10mm × 10 mm static simulation test porous structure models, as shown in Figure 5b.

### 2.2 Material and boundary condition settings

Definition of material properties: In the numerical simulation, the porous structure is made of Ti-6Al-4V alloy, and the compression disc material used in the analysis is Cr12 steel and the detailed material parameters are referred to in Table 1.

**Table 1:** Material parameters.

Material	Density	Elastic modulus	Poisson's ratio
i-6Al-4V	4,430 kg/m³	110 GPa	0.342
CR No. 12 steel	7,800 kg/m³	210 GPa	0.3

**Figure 6:**
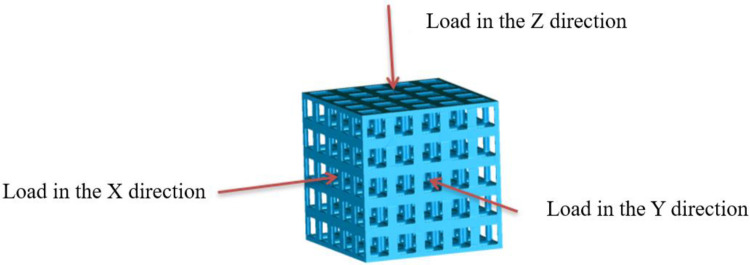
The direction in which the load is applied.

**Figure 7:**
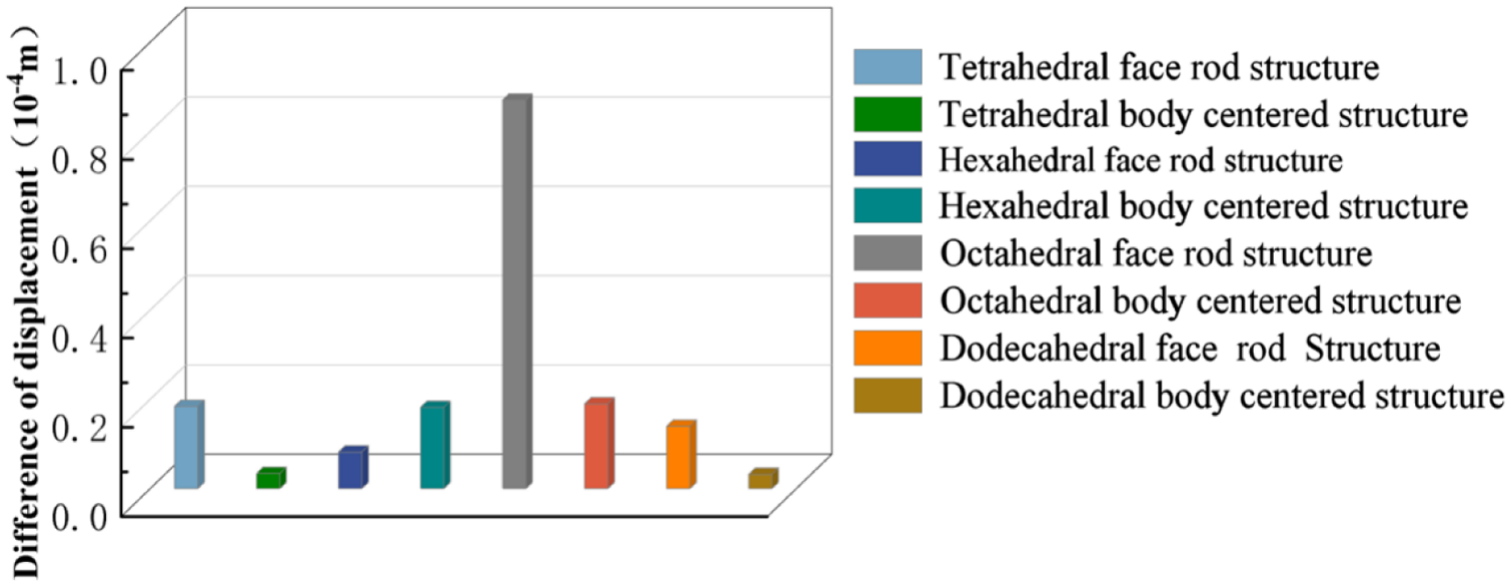
Numerical analysis results.

Isotropic analysis of loads and boundary conditions: During the analysis, fixed boundary conditions are set on the lower compression disc to simulate the constraint environment in the experiment. A compressive force of 50 N is applied in the analysis to simulate the load during the experiment. During the analysis, the compressive force is applied to the upper compression disc as a predetermined force, as shown in Figure 6. As shown in Table 2, the numerical analysis model compares and analyzes the three dimensions of the load applied by 6 degrees of freedom on the model of 8 types of porous structures of 10mm × 10mm × 10 mm, and compares the displacement contour and maximum stress contour of the 8 types of porous structures under compressive condition by observing the contour plots in Table 2, and takes the maximum interval values to make a histogram arranged in ascending order, as shown in Figure 7.

**Table 2:** Cloud map of compressive displacement for porous structure types.

Types of porous structures	X-axis direction	Y-axis direction	Z-axis direction
4-sided decent rod structure	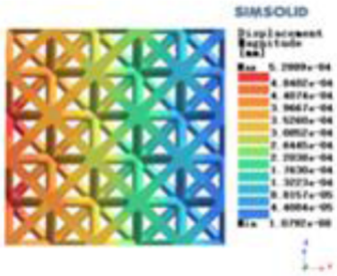	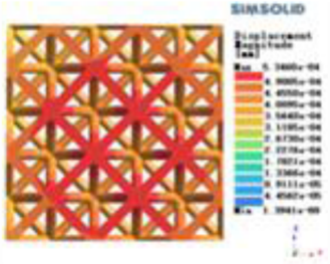	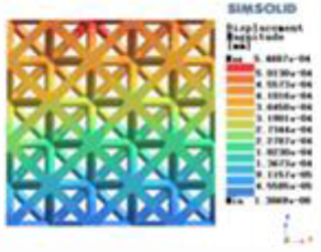
4-hedral body-centered structure	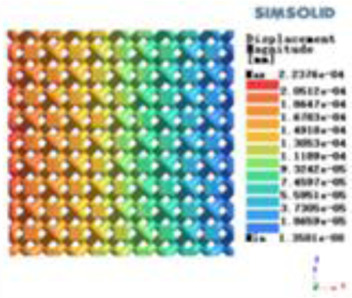	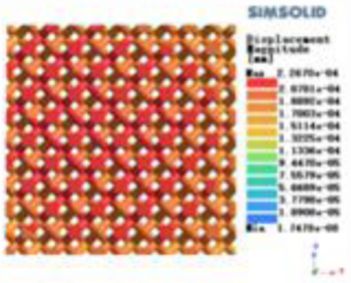	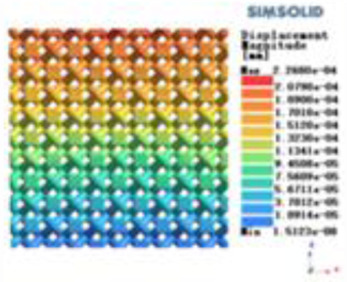
6-sided decent rod structure	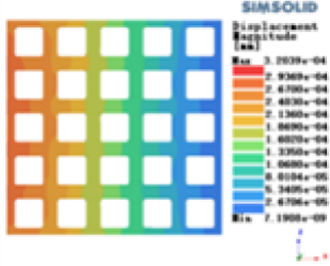	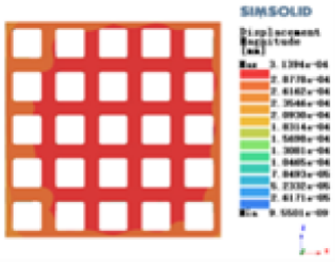	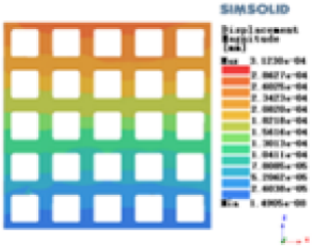
6-hedral body-centered structure	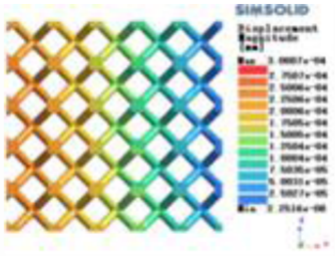	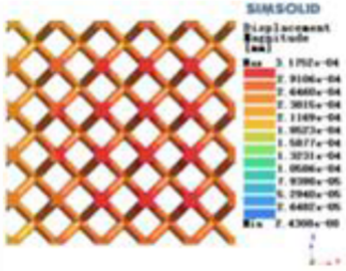	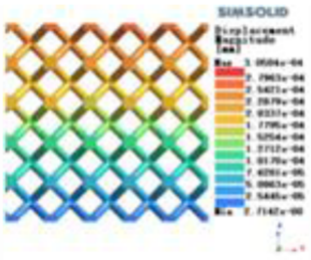
8-sided decent rod structure	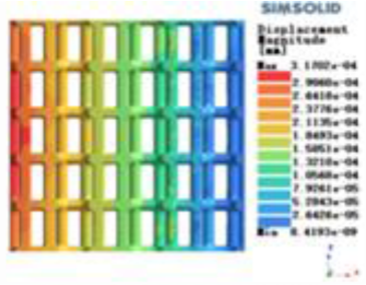	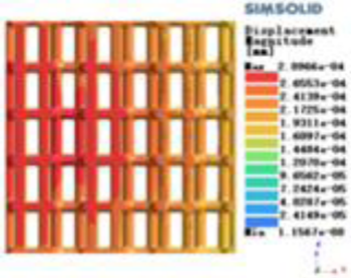	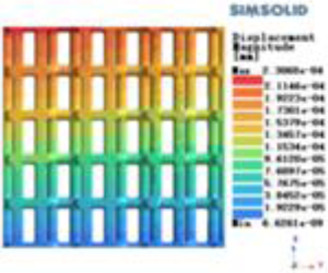
8-hedral body-centered structure	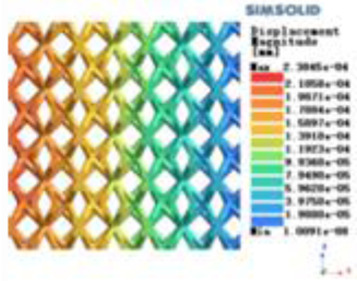	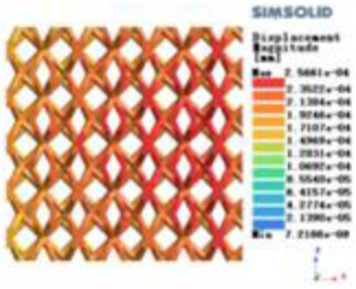	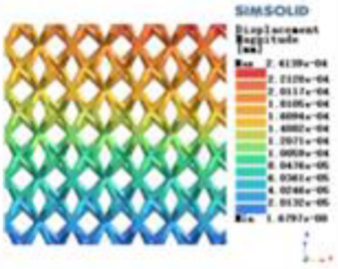
12-sided decent rod structure	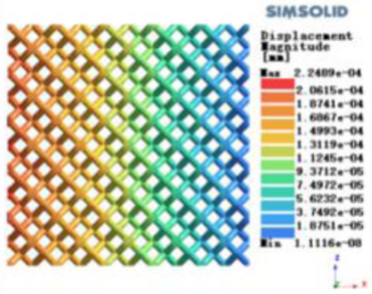	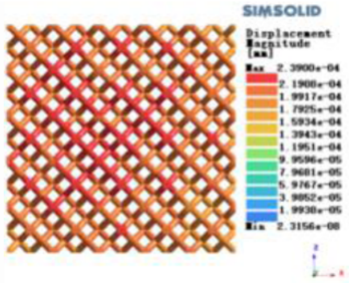	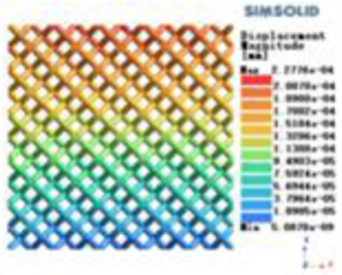
12-hedral body-centered structure	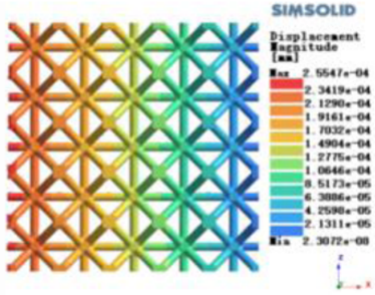	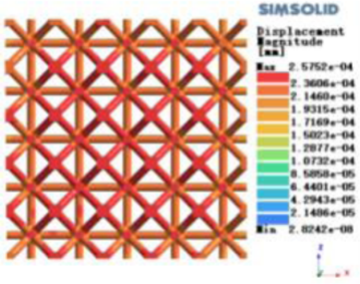	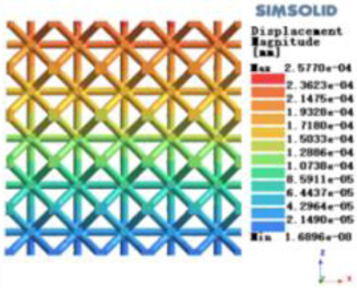

Modulus of elasticity analysis of loads and boundary conditions: During the analysis, fixed boundary conditions are set on the lower compression disc to simulate the constraint environment in the experiment. A compressive force of 5,000 N was applied in the analysis to simulate the load during the experiment. The compressive force is applied to the upper compression disc as a predetermined force, and the result is exported as a displacement contour as shown in Table 3. By observing the contour plots in Table 2, the differences in the displacements of 8 types of porous structures in 3 pairs of degrees of freedom were analyzed and histogrammed, as shown in section 2.3 “Numerical analysis results”.

**Table 3:** Cloud map of compressive for porous structure types.

Types of porous structures	Displacement contour diagram	Stress distribution contour diagram	Equivalent variable contour diagram	Strain energy density contour
4-sided defensible column structure	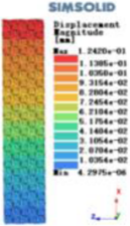	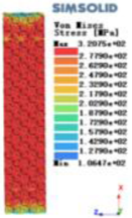	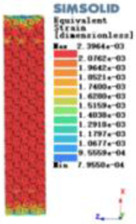	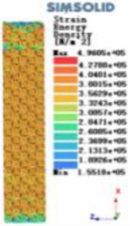
4-hedral body-centered structure	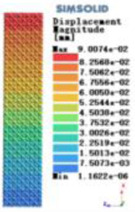	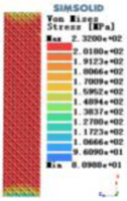	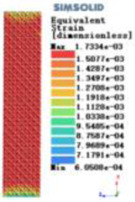	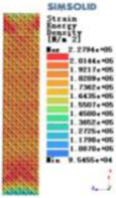
6-sided defensible column structure	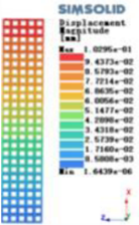	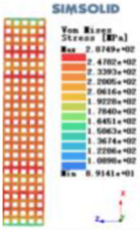	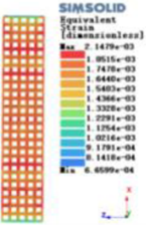	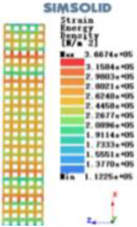
6 -hedral body-centered structure	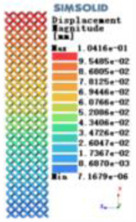	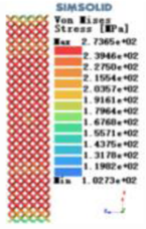	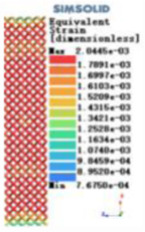	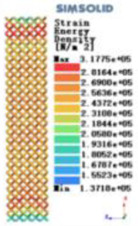
8-sided defensible column structure	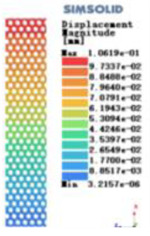	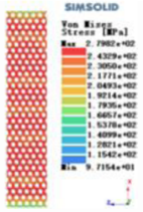	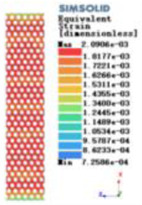	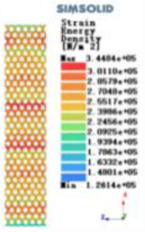
8-hedral body-centered structure	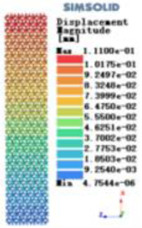	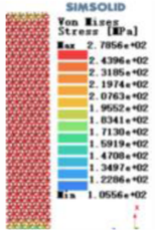	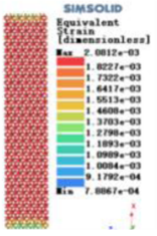	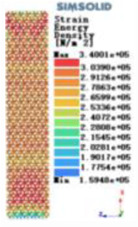
12-sided defensible column structure	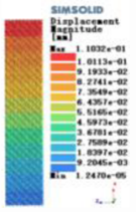	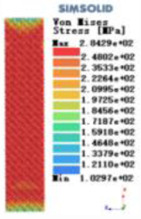	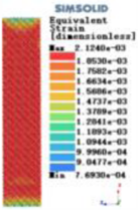	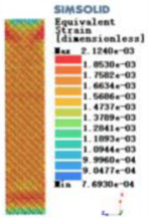
12-hedral body-centered structure	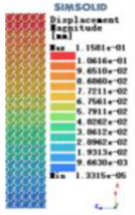	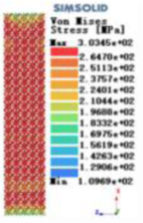	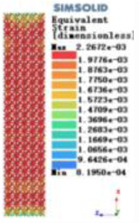	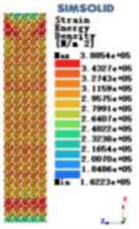

### 2.3 Numerical analysis results

As shown in Figure 7, for the above types of porous structures, the isotropy of the body-centered structure is more significant than that of the surface-column structure, but the octahedral face rod structure (honeycomb structure) is widely used in machinery, construction, and other fields. Studies have shown (18) that isotropic porous structures are more suitable than anisotropic porous structures as implantable prostheses. There are two reasons for this: firstly, it is difficult to have time to correct the orientation of the porous structure during the design process of porous implants, so the isotropic porous structure will buy patients more treatment time during the design of the prosthesis, and secondly, the prosthesis will face very complex working conditions in practical applications. In this case, the anisotropic porous structure is difficult to predict and has a lot of uncertainty, which is not conducive to the stability of the prosthesis *in vivo*. Therefore, isotropic porous structure, tetrahedral body-centered structure, and dodecahedral body-centered structure were preliminarily selected as the research objects of implantable prosthesis design.

The human femur needs to bear the weight of the upper body of the human body, and its main force is the vertical downward pressure in the vertical direction, so the implanted prosthesis that repairs the bone defect in this part should have a high equivalent elasticity and reduce the relative displacement of the implanted prosthesis and the surrounding bones in the longitudinal section. The density of titanium alloy is 4.51 g/cm^3^, which is much larger than the density of human bone (about 1.5 g/cm^3^), To make the unit mass of the implanted prosthesis close to that of human bone, it is necessary to lightweight design the implanted prosthesis, so the porous type with high equivalent elasticity should be selected to play a better supporting role.

As shown in Figure 5, under the same working conditions, the maximum displacement of the tetrahedral body center structure is significantly smaller than that of other types of porous structures, which is less than 12.4% of the temporary (6-sided surface column structure) and less than 27.5% of the maximum displacement (8-sided body center structure). The stress distribution of the tetrahedral body center structure is uniform, and the maximum stress value is significantly smaller than that of other types of porous structures. It shows that the tetrahedral body-center structure has good stability when it is subjected to pressure as an implanted prosthesis structure.

**Figure 8:**
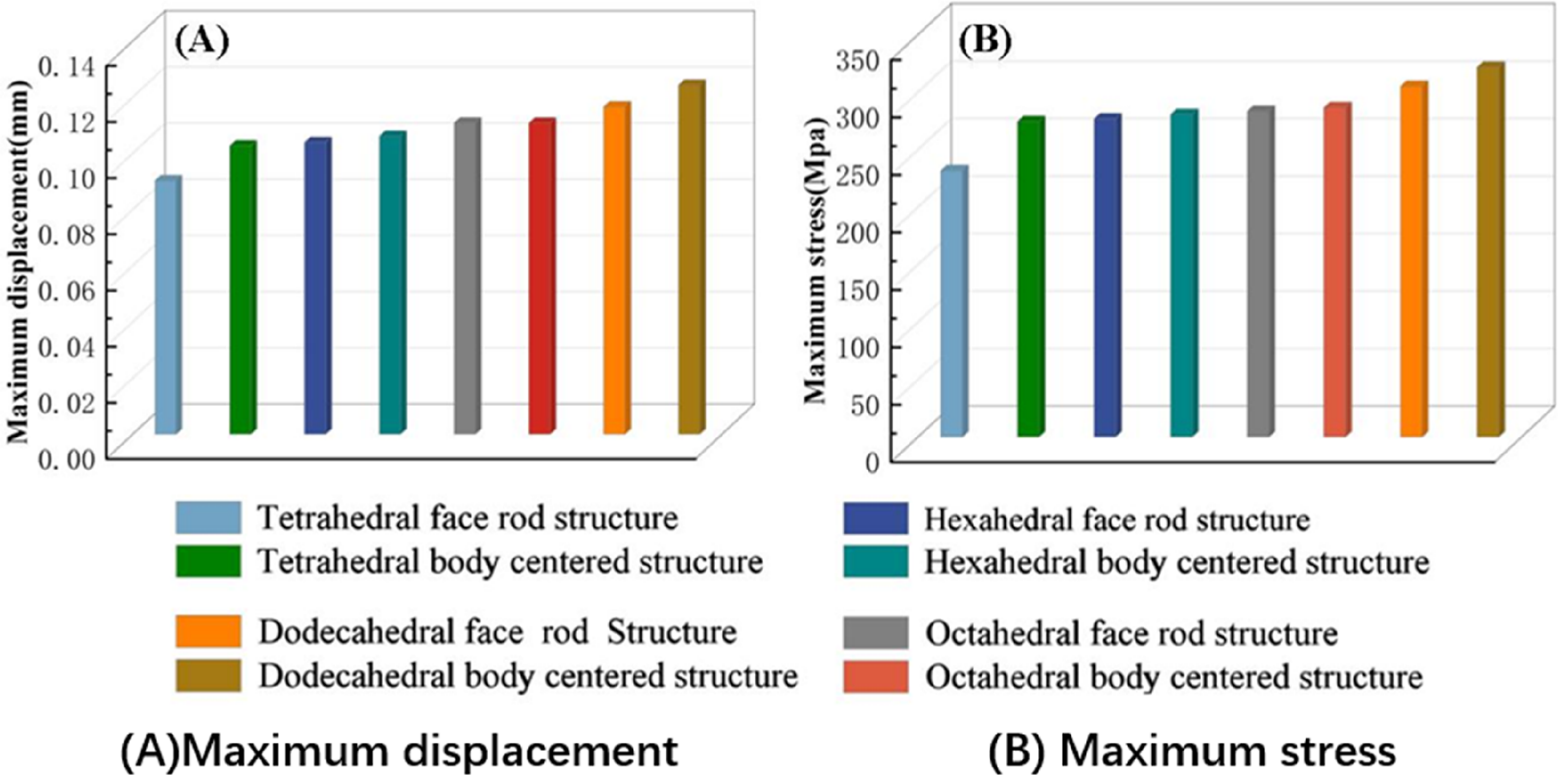
Numerical analysis results.

According to Maxwell's criterion, the equivalent elasticity of the tensile dominant structure is usually higher than that of the bending dominant structure, and the tetrahedral body-centered structure is a typical tensile dominant structure, and its equivalent elasticity in the elastic stage is better, which can be confirmed in Table 3; Figure 8. Although only the above eight porous structures are numerically analyzed, due to the representativeness and continuity of the selection, it can be speculated that with the increase of the number of faces, the porous structures will be more inclined to bend-dominant structures, so it is difficult to have porous structures with better equivalent elasticity than tetrahedral body-centered structures.

In summary, the tetrahedral body-centered structure has good isotropy and high equivalent elasticity at the same time, and is most suitable for the design of implantable prostheses among the 8 types of porous structures, in addition, the 6-hedral body-like column structure and the 12-hedral body-column structure also exhibit good isotropy and high equivalent elasticity, which can be used as an alternative to compare the machinability and bone ingrowth with the tetrahedral body-centered structure.

## 3 Construction of porous structure evaluation system

### 3.1 The overall structure of the evaluation system

**Figure 9:**
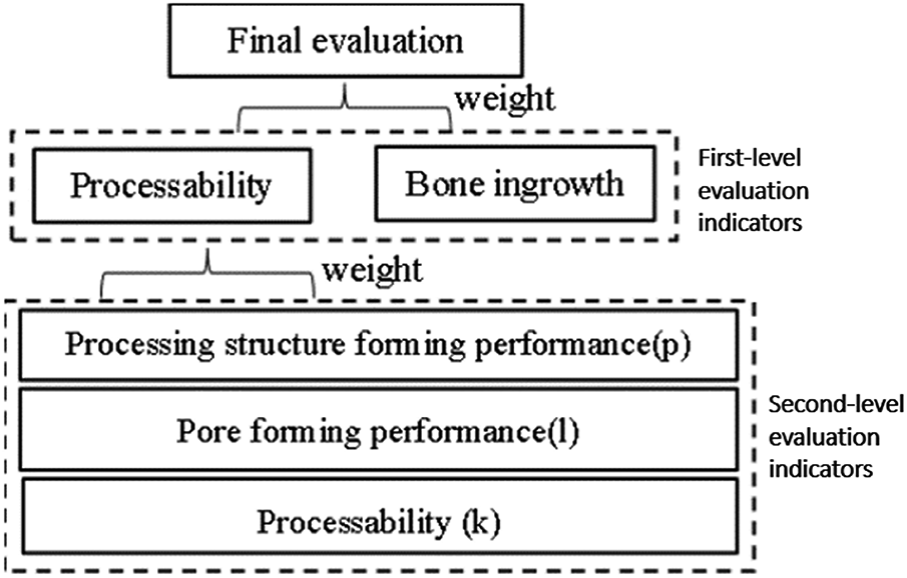
Evaluation system.

The porous types mentioned above mainly rely on the polyhedral structure to establish a porous model, but the types of porous structures in clinical research are not limited to this one, so it is necessary to analyze the types of porous structures commonly used in clinical practice. Through communication with 7 experts from Dalian Zhongshan Hospital, the First Affiliated Hospital of Dalian Medical University, the Affiliated Hospital of Beihua University, and Jilin Orthopedic Hospital, we were able to construct an evaluation system for implanted prostheses with porous structures. As shown in Figure 9, the evaluation system divides the evaluation indicators into two levels, which are synthesized into the practical clinical application reliability of the implanted prosthesis porous structure type.

### 3.2 Machinability evaluation index

To achieve personalized implantation of the defect area, the contour shape of the prosthesis needs to match the defective area of the human skeleton. Therefore, the most suitable processing method is 3D printing processing at present. When studying suitable porous structures, the feasibility of their processing needs to be considered. The evaluation value of machinability consists of three parts, namely: the molding performance of the processing structure, the pore formability, and the processing formability.

1. Molding performance of processing structure The evaluation method of the molding performance of the processed structure is to compare the numerical analysis and mechanical experiment results of the porous structure, and the fit between the two can reflect the machinability of the porous structure on the side, and the forming performance equation of the processed structure is defined as:
  (1) $$P=\left|\frac{E}{E^{\prime }-E}\right|$$
  (2) $$E=\frac{F}{S}$$
  (3) $$E=\frac{F"}{S^{\prime }}$$

Among them, P represents the molding performance of the processed structure, E represents the equivalent elasticity of the specimen in the experiment, F represents the load on the specimen in the experiment, S represents the displacement distance of the compression disc of the testing machine, E′ represents the equivalent elasticity of the model in the numerical analysis, F′ represents the load applied in the numerical analysis, and S’ represents the maximum displacement.

**Figure 10:**
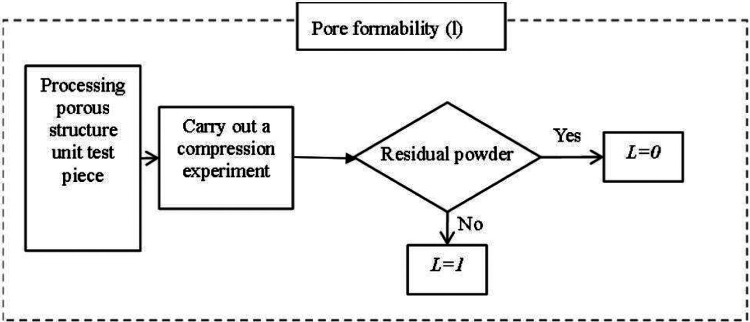
The process of evaluating manufacturability based on the pores of a porous structure.

**Figure 11:**
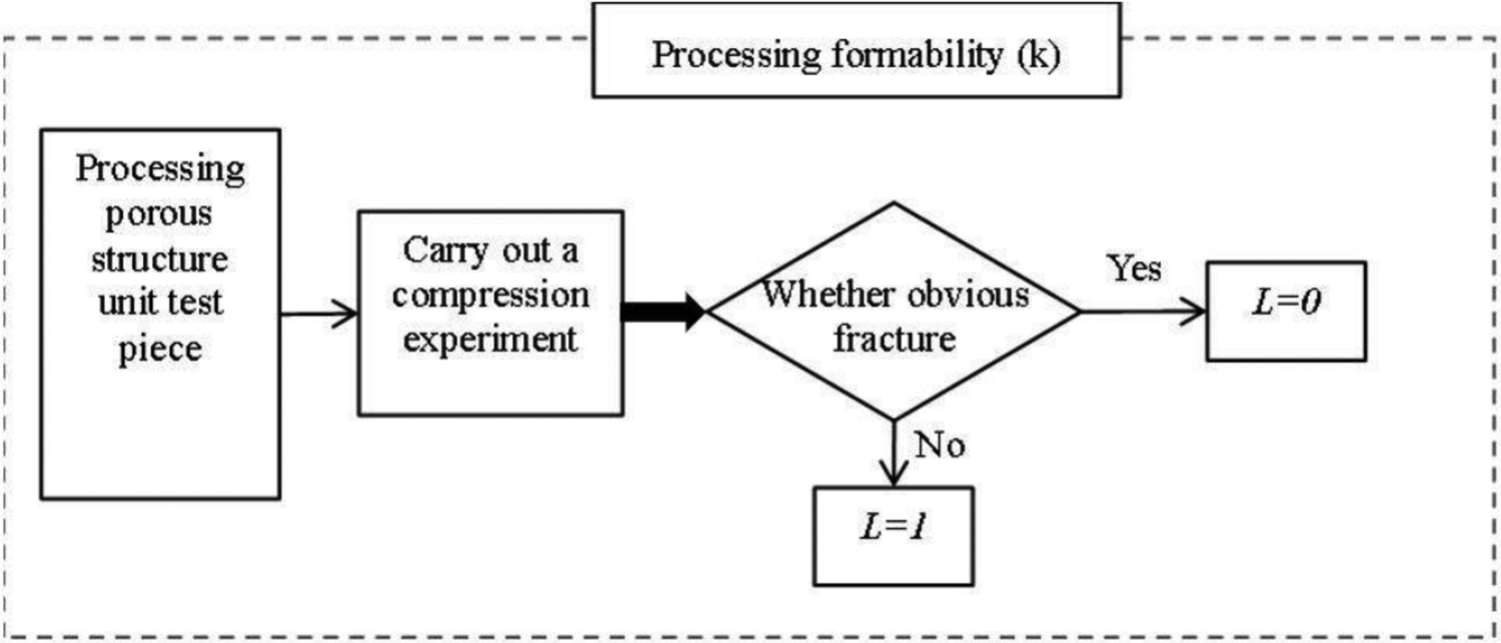
The process of evaluating manufacturability based on the beam structure.

2. Poremolding performance As shown in Figure 10, pore-forming performance refers to the pore uniformity of porous structure, too many small pore size pores after processing, the powder is difficult to clean, can spill after implantation, cause infection or inflammation. Therefore, it needs to be evaluated separately, and if this is the case, it is 0, otherwise it is 1.
3. Processing and molding performance The processing and molding performance of porous structures needs to meet the process requirements. A large number of beam structures are prone to fracture during 3D printing processing, resulting in large-scale fractures and debris splashes, which will cause damage and medical accidents if implanted in the body. Therefore, this situation needs to be evaluated separately, and if there is this case, it is 0, otherwise, it is 1, As shown in Figure 11.

### 3.3 Evaluation of bone ingrowth

For implanted prostheses that repair bone defects, whether the surrounding bones can grow to them after implantation is an important indicator to evaluate the porous structure. CT was used to obtain the gray value of the bone around the implanted prosthesis, and the grayscale change per unit of time was analyzed to evaluate whether the porous structure was conducive to bone growth.

(4) $$J=\frac{H_{u}}{T}$$

Among them, J represents the evaluation value of bone ingrowth, Hu represents the increase in gray value of bone around the implanted prosthesis, and T represents unit time.

### 3.4 Calculation method of evaluation index weight

Weight is an important part of the evaluation system, which directly affects the evaluation results. To evaluate the porous structure more accurately, combined Equation 4 to evaluate its bone ingrowth and hired seven experts to assess the weights. The evaluation criteria are shown in Table 4 and the evaluation results are shown in Tables 5, 6. The weight of the first-level indicator points is W1 = (0.42 0.58), and the weight of the second-level index points is W2 = (0.25 0.29 0.46).

**Table 4:** Evaluation criteria.

Score	0	0.1	0.2–0.4	0.5–0.7	0.8 or more
Grading Criteria	Not at all important	Not very important	More important	Very important	Other metrics can be ignored

**Table 5:** First-level evaluation indicators.

Evaluators	Machinability	Bone ingrowth
Specialist A	0.4	0.6
Specialist B	0.3	0.7
Specialist C	0.45	0.55
Specialist D	0.4	0.6
Specialist E	0.5	0.5
Specialist F	0.5	0.5
Specialist G	0.42	0.58

**Table 6:** Second-level evaluation indicators.

Evaluators	Molding properties of processed structures	Porosity formability	Processability and formability
Specialist A	0.2	0.3	0.5
Specialist B	0.3	0.3	0.4
Specialist C	0.4	0.2	0.4
Specialist D	0.3	0.3	0.4
Specialist E	0.23	0.42	0.35
Specialist F	0.1	0.3	0.6
Specialist G	0.2	0.2	0.6
Weight	0.25	0.29	0.46

## 4 Research on various evaluation indicators

### 4.1 Processability index of porous structure

#### 4.1.1 Purpose of mechanical compression experiment

To ensure the reliability of the research and the quality analysis of the actual processed porous structure, this paper adopts a variety of actually processed porous structures and compares the mechanical compression experiments to provide an accuracy guarantee.

#### 4.1.2 Mechanical compression test equipment

**Figure 12:**
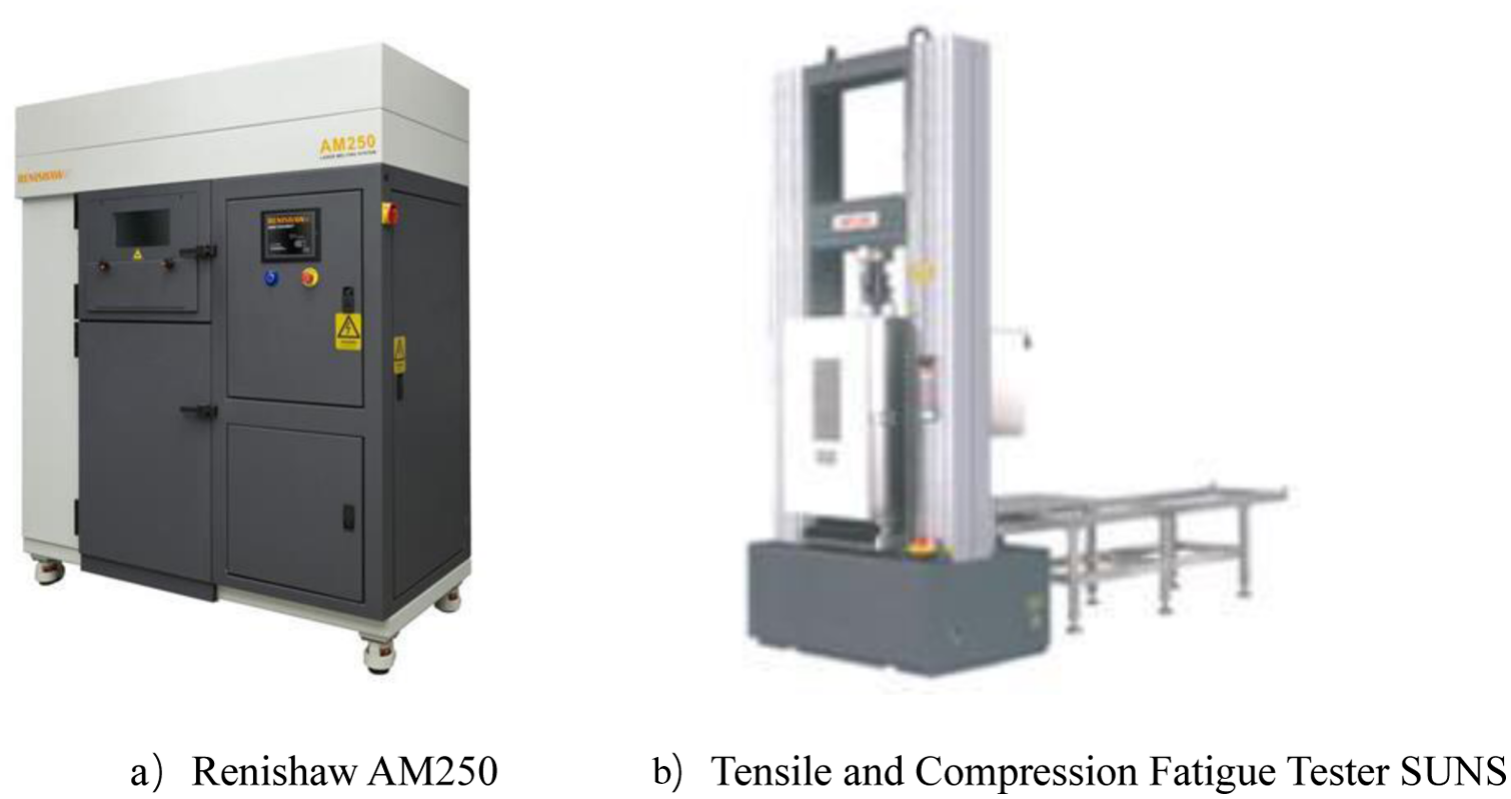
Experimental equipment.

As shown in Figure 12 Laser molten metal rapid prototyping machine AM250 (Renishaw, UK); Tensile Compression Fatigue Testing Machine (SUNS, Shenzhen, China)

#### 4.1.3 Experimental methods for mechanical compression

**Figure 13:**
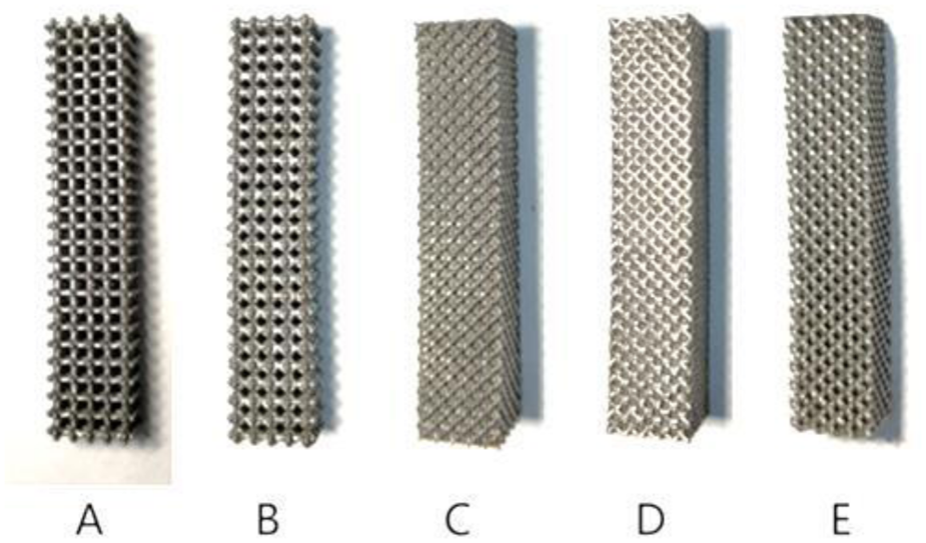
Different types of porous structure test samples.

In this study, titanium alloy (Ti-6Al-4V) powder with a particle size of 15–53 μm was used as the raw material, and the laser molten metal rapid prototyping machine AM250 was used to process five kinds of porous structure test samples, and the overall size and structural unit size of the five porous structures were the same (the overall dimensions were 10 mm × 10 mm × 50 mm, and the structural unit size was 2 mm × 2 mm × 2 mm), and the finished product was shown in the Figure 13. Structure A is a 6-sided column structure, Structure D is a 12-sided column structure, Structure E is a 4-sided body center structure, and Structure B and Structure C are two commonly used structures in clinical practice.

**Figure 14:**
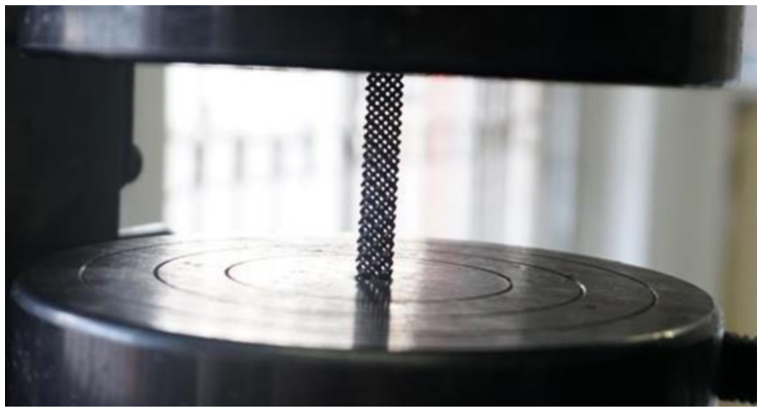
Process of compression experiments on specimens with porous structures.

As shown in Figure 14, to reduce the influence of chance on the results in the experiment, each group of specimens is 2, and all of them are processed using the same processing method, and the average value of the test results of the two specimens is used for analysis in the statistical data.

#### 4.1.4 Experimental results

**Figure 15:**
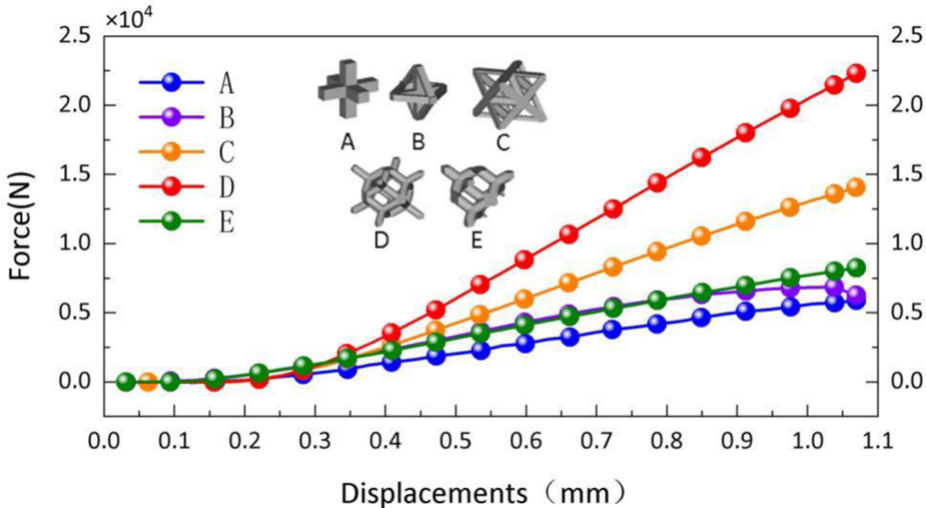
Compressing experimental data.

In the compression experiment, after the compression of the specimens of structures C and D, there is a large amount of residual raw material powder on the test bench. The specimens of structures A and B have obvious fracture sounds after being subjected to pressure, and debris splashing after permanent deformation. In contrast, structure E showed more stable mechanical properties in the compression test, and even after permanent deformation, it could continue to be compressed and did not fracture the supporting structure for a long period. The average test results of the two specimens in each porous structure were averaged and the relationship between force and displacement of each structure during the compression experiment was plotted, as shown in the Figure 15.

Structure B, structure C, and structure D have a variety of different pore sizes, and their minimum pore sizes are all relatively small (less than 0.6 mm). However, some residual powder may be generated during the processing of structures C and D, which is difficult to remove from the crevices of the porous structure. In contrast, structure B has a larger maximum pore size and fewer smaller pore size regions, so there is no powder residue. The machinability of structure E (4-hedral body-centered structure) is significantly better than that of other types of porous structures.

### 4.2 Indicators of bone ingrowth in porous structures

#### 4.2.1 Purpose of animal experiments

Based on the results of the analysis of the mechanical properties and machinability of the porous structure, the animal implantation experiment was carried out to ensure that the selected porous structure had good bone ingrowth.

#### 4.2.2 Experimental equipment and objects

**Figure 16:**
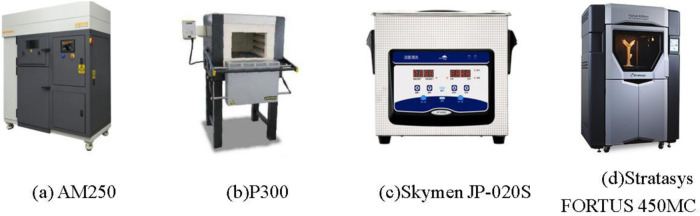
Experimental equipment.

The experimental subjects selected in this paper are 1-year-old healthy adult male beagles weighing 10 kg; The experimental instruments used are: laser molten metal rapid prototyping machine AM250 (Renishaw company, UK) Muffle furnace P300 (Nabertherm Industrial Furnace GmbH, Germany) Radiography computed tomography equipment ScintCare CT16X (Zhejiang Mingfeng Medical System Co., Ltd., China) Ultrasonic Cleaner Skymen JP-020S (China Guangdong Tianmen Cleaning Equipment Shenzhen Co., Ltd.) Digital photographic x-ray machine RayNova DRsc4 (Liaoning Kaipu Medical System Co., Ltd., China) Scanning Electron Microscope SU3500 (Hitachi Manufacturing Co., Ltd.) Figure 16 shows experimental equipment.

#### 4.2.3 Experimental methods

**Figure 17:**
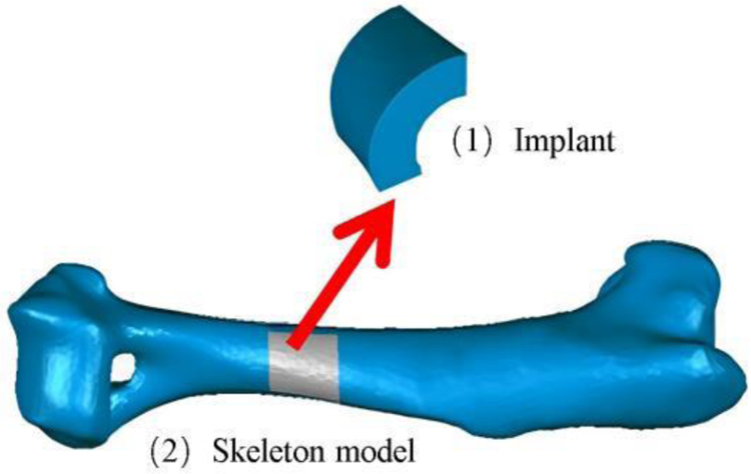
The models of implanted prosthesis.

**Figure 18:**
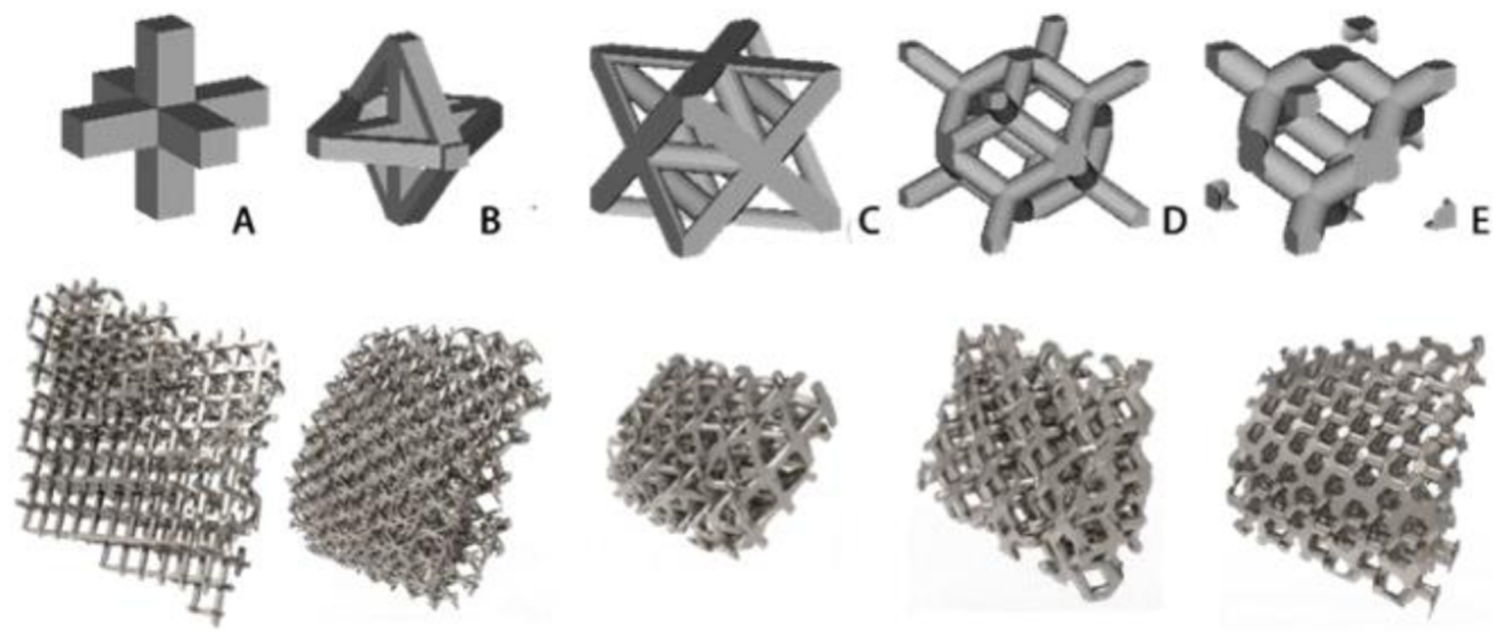
Porous interstitial treatment of implant model.

**Figure 19:**
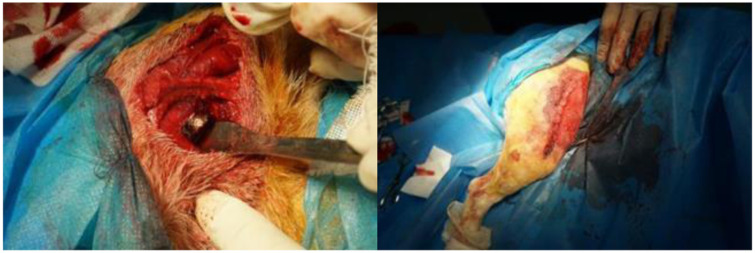
The process of animal experiments.

1. Establish a skeletal model Firstly, the three-dimensional skeleton model of the experimental dog was established through reverse engineering, and the processing of the prosthesis was carried out based on this model, which was conducive to the implantation of the prosthesis and the skeleton of the experimental dog.
2. Implant prosthesis A block with a projection area of 1 cm^2^ was intercepted from the bone to be implanted as a block model of the implanted prosthesis (Figure 17), and the influence of irrelevant factors such as the poor integration between the implanted prosthesis and the surrounding bone was excluded, to better verify the bone ingrowth of many different types of porous structures.
3. Multi-porous implant prosthesis model Each porous structure has the same element structure size and similar support rod radius. The treated porous implant prosthesis model and the corresponding porous structure are shown in Figure 18.
4. Treatment of implanted prostheses The implant is annealed and cleaned with a Skymen JP-020S ultrasonic cleaner to reduce the stress effects of processing, remove residues, and ensure the stability of the implant.
5. Implantation surgery. This experiment was performed in the animal laboratory of Beihua University, Jilin Province, China, in accordance with the ARRIVE guidelines, and in accordance with the UK Animals (Scientific Procedures) Act 1986 and related guidelines, and the EU Directive 2010/63/EU. This study was approved by the Medical Ethics Review Committee of Beihua University. The prosthesis was aseptically treated by high temperature and autoclave sterilization, general anesthesia was followed during the operation, aseptic conditions and analgesic procedures were followed, the incision corresponded to the bone gap, 2 cm in size, the subcutaneous tissue and periosteum were separated and the periosteum was sutured, and the postoperative intravenous infusion of clindamycin phosphate was antibacterial and anti-inflammatory. The results were well observed, with no symptoms of infection, and the postoperative animal activity, diet, and healing of the surgical area were good. Figure 19 shows part of the experimental process.
6. Postoperative data collection x-ray and CT scans were performed weekly on the experimental dogs for 1–8 weeks after surgery to record the bone growth around the porous structure.

#### 4.2.4 Experimental results

**Figure 20:**
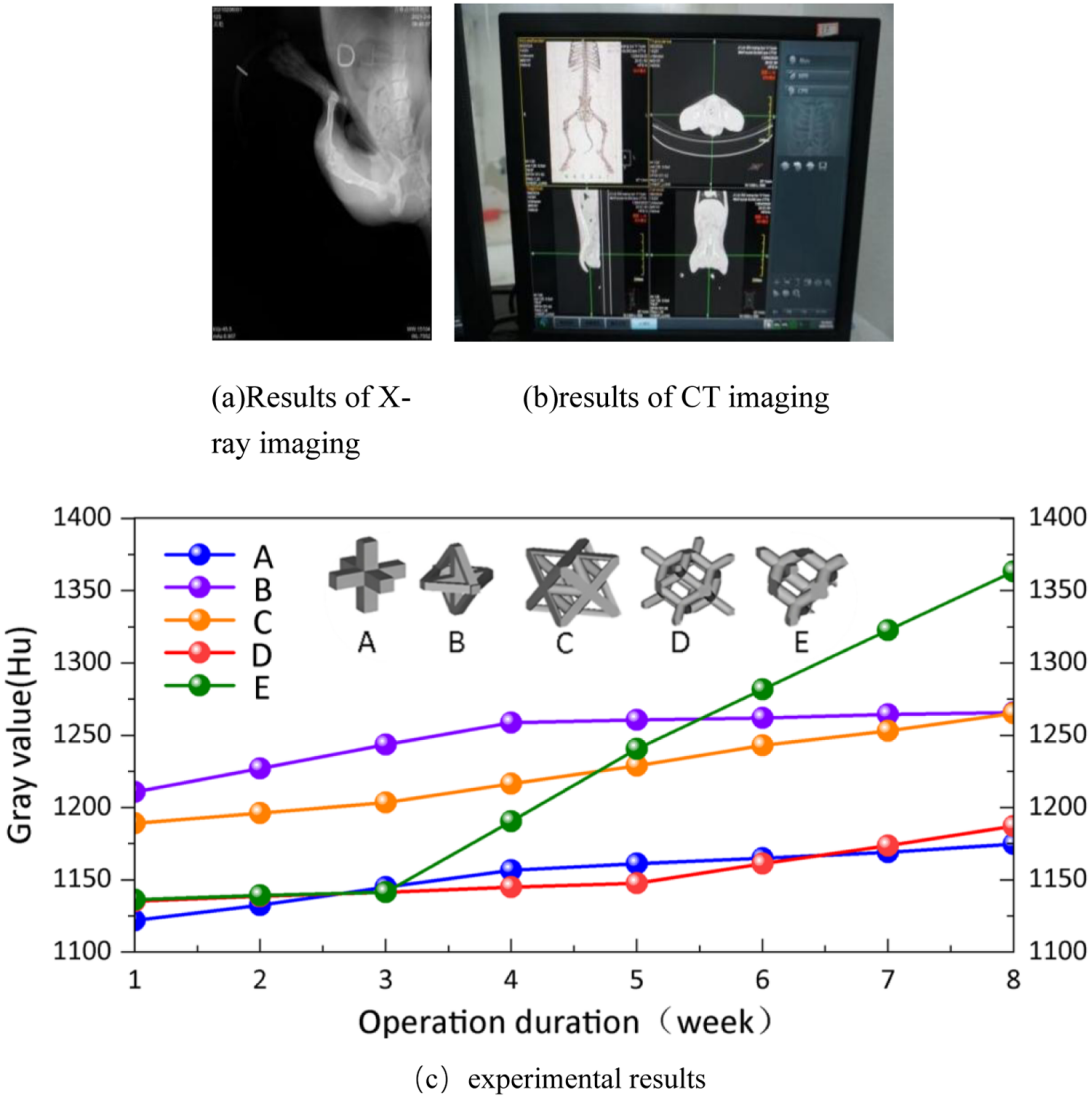
The results of animal experiments.

In Figure 20(a,b) represent the results of x-ray and CT imaging respectively. Quantitative analysis of the bone ingrowth of various types of porous structures is conducive to the selection of the porous structure of the implanted prosthesis. On the bone model obtained from each CT scan, 10 points are randomly selected at the positions connected to each implanted prosthesis, and the gray values of these points are acquired. Then, the average values are calculated for comparative analysis. The experimental dogs showed good tolerance to the implanted prostheses, and no infections in soft or hard tissues were observed in all the wounds after the surgery. At the 8th week after the surgery, no implant loss occurred. The average gray values of the bone tissues around the implants in the experimental group and the control group were plotted as a curve, as shown in (c) of Figure 20.

Analyze according to Figure 20(c). Long-term stability of implanted prostheses requires not only mechanical and shape matching, but also stable fixation between the bone-scaffold interface (19). Therefore, it is crucial to achieve a balance between bone tissue growth and mechanical support for weight-bearing bone defect repair. Studies on periprosthetic bone tissue growth in experimental dogs have found that there are differences in the gray values of various types of implanted periprosthetic bones. The gray value of the bone tissue around the implant showed a clear upward trend, while the growth rate of porous structure A was slower, and saturation began to appear in the fourth week. The porous structure B showed that the gray value of bone tissue even showed a slow downward trend from the fourth week. In porous structure C, the bone tissue around the implant starts to grow significantly in the third week, but the growth rate is much slower than that around the implant in porous structure E. The growth rate of structure D is comparable to that of porous structure C, and growth begins to appear in the fifth week. By the eighth week after surgery, the bone tissue surrounding the implanted prosthesis in porous structures A and B had stalled, while the porous structures C and D were still growing, but at a slower rate than in porous structures E. Structures A and B have high porosity and promote bone ingrowth. Still, there are processing defects that adversely affect the growth of the bone around the implanted prosthesis. The porosity of Structures D and E is similar, but the pore size of Structure E is larger, so the bone ingrowth of Structure E is better than that of Structure D in the later rehabilitation process. This is consistent with (20, 21) studies. The porous structure C has low porosity and uneven pore size, which may be the main reason for its bone growth.

## 5 Evaluation results

### 5.1 Methods for calculating the evaluation value of porous structure

According to the evaluation system of porous structure, combined with the experts' scores on the weight of the evaluation index, the calculation method of the evaluation value of porous structure was determined. The evaluation of machinability includes three evaluation indexes, and the calculation equation is as follows:

(5) $$\begin{array}{rl} & I=\mathrm{rlP}+r2L+r3K \end{array}$$

Among them, I represents the evaluation value of machinability, *P* represents the molding performance of the processing structure, L represents the evaluation value of pore formability, K represents the evaluation value of processing formability, and r1, r2, and r3 are the corresponding weights of each evaluation index. According to the evaluation system, the final evaluation index of porous structure is calculated, and the calculation equation is as follows:

(6) W=c1I+c2J

W is the final evaluation value, I is the machinability evaluation value, J is the bone ingrowth evaluation value, and c1 and c2 are the corresponding weights of each evaluation index.

### 5.2 Calculation of machinability evaluation value

According to the results of numerical analysis and compression experiments, the evaluation of the machinability of porous structures was completed by combining Equations (1–3, 5); Figures 10, 11, as shown in Table 7.

**Table 7:** The evaluation of machinability.

Structure type	Structure A	Structure B	Structure C	Structure D	Structure E
Molding properties of processed structures	0.18	0.688	0.258	0.92	1.1
Porosity formability	0.29	0.29	0	0	0.29
Porosity formability	0	0	0.46	0.46	0.46
Machinability	0.47	0.978	0.718	1.38	1.85

It can be found from the table that the machinability of structure E (4hedral body-centered structure) is significantly better than that of other types of porous structures, which proves that the porous structure is suitable for processing by 3D printing and has good applicability for the design of personalized implanted prostheses.

### 5.3 Calculation of the evaluation value of bone ingrowth

Because 1–2 weeks after surgery is the hematoma organization period, the bone growth is slow at this time, and even the possibility of gray value decline from the gray value is observed, so it is Structure type Structure A Structure B Structure C Structure D Structure E Molding properties of processed structures 0.18 0.688 0.258 0.92 1.1 Porosity formability 0.29 0.29 0 0 0.29 Porosity formability 0 0 0.46 0.46 0.46 Machinability 0.47 0.978 0.718 1.38 1.85 not used as the basis for judging bone ingrowth, and 3 weeks after surgery is the original callus formation period and callus transformation and shaping period. It is more convincing to choose the change of gray value at this time as the basis for judging bone ingrowth of porous structure. The gray value growth rate was used as the criterion for the evaluation of bone ingrowth of five porous structures, as shown in Table 8.

**Table 8:** The evaluation of bone ingrowth.

Structure type	Structure A	Structure B	Structure C	Structure D	Structure E
Average growth rate [Hu/day]	0.76	0.65	1.67	1.03	5.11
Bone growth into the evaluation value	0.44	0.38	0.97	0.60	2.96

### 5.4 Porous structure evaluation results

Equations 5, 6; Tables 4, 5, 8 are used to calculate the final evaluation values for the five porous structure types as shown in Table 9.

**Table 9:** The final evaluation value of the porous structure.

Structure type	Structure A	Structure B	Structure C	Structure D	Structure E
Final evaluation value	0.91	1.358	1.688	1.98	4.81

According to Table 7, it can be seen that the composite score of porous structure E (tetrahedral body-centered structure) is significantly better than that of other structures among various porous structure types. According to the above, the structure exhibits superior properties to other types in terms of isotropy, equivalent elasticity, machinability, and bone ingrowth, which proves that the structure is suitable for research as a type of porous structure for implanted prosthesis.

## 6 Conclusion

Given the difference in the degree of different porous structures to reduce the stress masking phenomenon, this study evaluated the mechanical properties, processing properties, and osseointegration properties of various types of porous structures by combining numerical analysis, mechanical experiments, and animal experiments based on the method of constructing an evaluation system, and drew the following conclusions:

1. In terms of structural mechanical properties, the 4-dihedral body core structure and the 12-hedral body center have good isotropy, which is very suitable for the design of implanted prostheses. Structure type Structure A Structure B Structure C Structure D Structure E Average growth rate [Hu/day] 0.76 0.65 1.67 1.03 5.11 Bone growth into the evaluation value 0.44 0.38 0.97 0.60 2.96 Structure type Structure A Structure B Structure C Structure D Structure E Final evaluation value 0.91 1.358 1.688 1.98 4.81
2. Compared with other polyhedral structures, the 4-dihedral body structure exhibits superior mechanical properties and machinability, making it suitable as a type of porous structure for implantable prosthesis design. The mechanical properties and machinability of the 4-dihedral body structure not only meet clinical needs but also improve the precision and success rate of surgery.
3. The tetrahedral body-core structure demonstrates excellent bone ingrowth, with the growth rate of bone around the implanted prosthesis being 3.38–4.4 times that of other types of porous structure implanted prostheses. This indicates that the 4-dihedral body structure can significantly promote bone regeneration and accelerate patient recovery.
4. In the newly established evaluation system, the final evaluation index of the 4-dihedral body-centered structure is significantly better than that of other types of porous structures, making it more suitable for the types of porous structures commonly used in clinical practice. Its final evaluation value of 4.81 is far superior to other types of structures.

Combining orthopedic clinical experience and existing literature reviews, the 4-dihedral body-centered structure shows significant advantages in reducing the stress masking effect, promoting bone growth, and improving treatment outcomes. The 4-dihedral body structure can effectively mimic the mechanical environment of natural bone, reducing the risk of prosthesis loosening and improving long-term stability. Additionally, the good machinability of this structure allows for customization based on the specific needs of patients, enhancing surgical precision and success rates.

In clinical applications, the 4-dihedral body-centered structure is particularly suitable for the repair of segmental bone defects. For example, in long bone defects or spinal fusion surgeries, using 4-dihedral body-centered structure implants can significantly improve treatment outcomes, reduce postoperative complications, and accelerate patient recovery. However, any implant design must also consider factors such as material biocompatibility, infection risk, and long-term biological reactions.

Therefore, future research directions should include larger sample size clinical trials to further validate the effectiveness and safety of the 4-dihedral body-centered structure in different clinical scenarios. Additionally, exploring the application of composite materials and the combination of growth factors or stem cell technology may further enhance the bone integration and functional recovery capabilities of implants.

In summary, this study ultimately selected the 4-dihedral body-centered structure as the type of porous structure for the design of implanted prostheses. Its advantages in reducing the stress masking effect, promoting bone growth, and improving treatment outcomes are of great significance for the clinical treatment of segmental bone defects.

## Data Availability

The original contributions presented in the study are included in the article/Supplementary Material, further inquiries can be directed to the corresponding author.

## References

[1] Vidal L Kampleitner C Brennan MÁ Hoornaert A Layrolle P. Reconstruction of large skeletal defects: current clinical therapeutic strategies and future directions using 3D printing. Front Bioeng Biotechnol. (2020) 8:61. 10.3389/fbioe.2020.00061 32117940 PMC7029716

[2] Martinez-Marquez D Delmar Y Sun S Stewart RA. Exploring macroporosity of additively manufactured titanium metamaterials for bone regeneration with quality by design: a systematic literature review. Materials (Basel). (2020) 13(21):4794. 10.3390/ma13214794 33121025 PMC7662257

[3] Donnaloja F Jacchetti E Soncini M Raimondi MT. Natural and synthetic polymers for bone scaffolds optimization. Polymers (Basel). (2020) 12:905. 10.3390/polym12040905 32295115 PMC7240703

[4] Jung A Jang J Ban HY Kim HJ Gweon B Lim D. Enhanced biomechanical and biological performance of titanium scaffolds with gradient in pore sizes. J Mater Res Technol. (2025) 34. 10.1016/j.jmrt.2024.12.216

[5] Wu S Liu X Yeung KWK Liu C Yang X. Biomimetic porous scaffolds for bone tissue engineering. Mater Sci Eng R Rep. (2014) 80:1–36. 10.1016/j.mser.2014.04.001

[6] Scheinpflug J Pfeiffenberger M Damerau A Schwarz F Textor M Lang A Journey into bone models: a review. Genes (Basel). (2018) 9:247. 10.3390/genes9050247 29748516 PMC5977187

[7] Chlebus E Kuznicka B Kurzynowski T Dybała B. Microstructure and mechanical behavior of ti-6Al-7Nb alloy produced by selective laser melting. Mater Charact. (2011) 62(5):488–95. 10.1016/j.matchar.2011.03.006

[8] Han C Yan C Wen S Xu T Li S Liu J Effects of the unit cell topology on the compression properties of porous Co-Cr scaffolds fabricated via selective laser melting. Rapid Prototyp J. (2017) 23(1):16–27. 10.1108/RPJ-08-2015-0114

[9] Xiao Z Yang Y Xiao R Bai Y Song C Wang D. Evaluation of topology-optimized lattice structures manufactured via selective laser melting. Mater Des. (2018) 143:27–37. 10.1016/j.matdes.2018.01.023

[10] Zhang L-C Liu Y Li S Hao Y. Additive manufacturing of titanium alloys by electron beam melting: a review. Adv Eng Mater. (2018) 20(5):1–16. 10.1002/adem.201700842

[11] Sarker A Leary M Fox K. Metallic additive manufacturing for bone-interfacing implants. Biointerphases. (2020) 15(5):050801. 10.1116/6.0000414 32942863

[12] Li J Cui X Hooper GJ Lim KS Woodfield TBF. Rational design, bio-functionalization and biological performance of hybrid additive manufactured titanium implants for orthopedic applications: a review. J Mech Behav Biomed Mater. (2020) 105:103671. 10.1016/j.jmbbm.2020.103671 32090892

[13] Hazlehurst KB Wang CJ Stanford M. A numerical investigation into the influence of the properties of cobalt chrome cellular structures on the load transfer to the periprosthetic femur following total hip arthroplasty. Med Eng Phys. (2014) 36(4):458–66. 10.1016/j.medengphy.2014.02.008 24613500

[14] Jianfeng K Ling W Changning S Zhongming J. Microstructure design for 3D printing of variable modulus metal prostheses. J Mech Eng. (2017) 53(5):175–80. 10.3901/JME.2017.05.175

[15] Rahyussalim AJ Marsetio AF Saleh I Kurniawati T Whulanza Y. The needs of current implant technology in orthopaedic prosthesis biomaterials application to reduce prosthesis failure rate. J Nanomater. (2016) 2016:1–9. 10.1155/2016/5386924

[16] Fu J Ni M Chen JY Deng T Guan HT Jia CQ Biocompatibility and biomechanics of personalized 3D-printed porous titanium reinforcement blocks for the reconstruction of severe acetabular bone defects. Chin J Orthop Surg. (2018) 26(10):945–50. 10.3977/j.issn.1005-8478.2018.10.18

[17] Marques A Miranda G Silva F Pinto P Carvalho Ó. Review on current limits and potentialities of technologies for biomedical ceramic scaffold production. J Biomed Mater Res Part B Appl Biomater. (2020) 109:377–93. 10.1002/jbm.b.34706 32924277

[18] Luxner MH Stampfl J Pettermann HE. Numerical simulations of 3D opencell structures—influence of structural irregularities on elastoplasticity and deformation localization. Int J Solids Struct. (2007) 44(9):2990–3003. 10.1016/j.ijsolstr.2006.08.039

[19] Fan HQ Wang FY He R Chen X Yang L. Preparation of 3D printed porous tantalum metal acetabular bone defect prosthesis and its preliminary clinical application. J Army Med Univ. (2022) 44(15):1516–22. 10.16016/j.2097-0927.202112205

[20] Wang C Xu D Lin L Li S Hou W He Y Large-pore-size Ti6Al4V scaffolds with different pore structures for vascularized bone regeneration. Mater Sci Eng C. (2021) 131:112499. 10.1016/j.msec.2021.112499 34857285

[21] Wang X Nie Z Chang J Lu ML Kang Y. Multiple channels with interconnected pores in a bioceramic scaffold promote bone tissue formation. Sci. Rep. (2021) 11:20447. 10.1038/s41598-021-00024-z 34650074 PMC8516977

